# Estimating the cost-savings associated with bundling maternal and child health interventions: a proposed methodology

**DOI:** 10.1186/1471-2458-13-S3-S27

**Published:** 2013-09-17

**Authors:** Adebiyi Adesina, Lori A Bollinger

**Affiliations:** 1 Futures Institute, 41-A New London Turnpike, Glastonbury, CT 06033, USA

## Abstract

**Background:**

There is a pressing need to include cost data in the Lives Saved Tool (LiST). This paper proposes a method that combines data from both the WHO CHOosing Interventions that are Cost-Effective (CHOICE) database and the OneHealth Tool (OHT) to develop unit costs for delivering child and maternal health services, both alone and bundled.

**Methods:**

First, a translog cost function is estimated to calculate factor shares of personnel, consumables, other direct (variable or recurrent costs excluding personnel and consumables) and indirect (capital or investment) costs. Primary source facility level data from Kenya, Namibia, South Africa, Uganda, Zambia and Zimbabwe are utilized, with separate analyses for hospitals and health centres. Second, the resulting other-direct and indirect factor shares are applied to country unit costs from the WHO CHOICE unit cost database to calculate those portions of unit cost. Third, the remainder of the costs is calculated using default data from the OHT. Fourth, we calculate the effect of bundling services by assuming that a LiST intervention visit takes an average of 20 minutes when delivered alone but only incremental time in addition to the basic visit when delivered in a bundle.

**Results:**

Personnel costs account for the greatest share of costs for both hospitals and health centres at 50% and 38%, respectively. The percentages differ between hospitals and health centres for consumables (21% versus 17%), other direct (7.5% versus 6.75%), and indirect (22% versus 23%) costs. Combining the other-direct and indirect factor shares with the WHO CHOICE database and the other costs from OHT provides a comprehensive cost estimate of LiST interventions. Finally, the cost of six recommended antenatal care (ANC) interventions is $69.76 when delivered alone, but $61.18 when delivered as a bundle, a savings of $8.58 (12.2%).

**Conclusions:**

This paper proposes a method for estimating a comprehensive cost of providing child and maternal health interventions by combining labor, consumables and drug costs from OHT with indirect and other-direct proportional costs from WHO CHOICE. In addition, we demonstrate the potential cost savings that can be achieved from bundling the delivery of essential antenatal care interventions rather than delivering the same interventions alone.

## Background

Integrating health care services is hypothesized to result in both cost savings and better outcomes [1]. Cost savings are likely to occur as the use of physical space is better managed, patients save time (and money) by attending an integrated facility, and medical records can be standardized and shared more easily. For example, integrating maternal and child health services with Human Immunodeficiency Virus (HIV) services is hypothesized to lead to more people, including children, learning their HIV status and thus beginning treatment appropriately as well as practicing safer sex and being treated for sexually transmitted infections (STI). The improved use of preventive and curative interventions should lead to better outcomes. Empirical evidence supports these hypotheses: a recent study from Zambia has shown that integrating antiretroviral therapy (ART) into antenatal care (ANC) clinics doubled uptake of ART in pregnant women. In addition the uptake of reproductive health services increased at the same rate as uptake of ART in facilities that offered integrated services [2]. Another study in Bangladesh found that integrating services resulted in a significant reduction in perinatal mortality [3]. An updated review of reports evaluating the impact of integrating immunization with other maternal and child health interventions found that coverage of the other interventions increased, although not to the same level as the original immunization coverage rates [4]. A recent Cochrane review, however, found mixed evidence on the impact of integrating services; although there was some evidence that utilization and outputs improved, no significant impact was found on overall health status [5].

The Lives Saved Tool (LiST) is a computer program developed by Futures Institute, Johns Hopkins University and the Child Health Epidemiology Reference Group to estimate the impact of scaling up child and maternal health interventions. It is used to project the future annual number and rate of child and maternal deaths up to 100 years. LiST can stratify these projections by cause of death as well as by health interventions. These projections are used in policy presentations to enhance knowledge of child and maternal health issues among policymakers and to build support for effective prevention and care interventions.

The LiST program is currently being augmented to include a mechanism for estimating the cost of providing child and maternal health interventions, as well as a mechanism for costing integrated, or “bundled,” services. The main cost-savings associated with bundling services is hypothesized to be from a reduction in indirect and labor costs, but consistent estimates of these costs across countries and interventions do not exist. Here we adapt data from both the WHO CHOosing Interventions that are Cost-Effective (CHOICE) database [6] and the OneHealth Tool (OHT) [7] to develop unit costs for delivering child and maternal health services, delivered both alone and bundled.

## Methods

We use an ingredients-based costing approach to calculate the various components of unit cost. The equation for calculating the unit cost of a LiST intervention is assumed to be the sum of five ingredients:

Equation 1: Unit Cost = Personnel + Drugs + Consumables + Other-direct + Indirect

**Figure 1:**
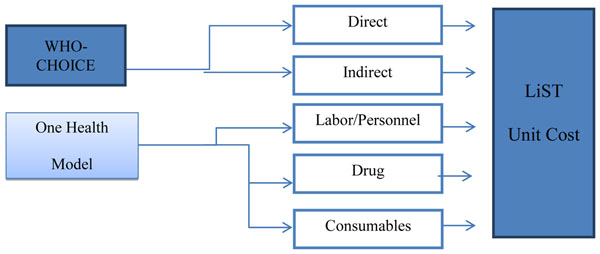
Schematic model and data sources for LiST costing

There are two main sources for the cost data utilized in calculating the unit costs. The WHO CHOICE database provides the “hotel” portion of cost per hospital day (i.e., excluding the cost of drugs and laboratory tests) by hospital level, as well as the hotel portion of the cost of outpatient visits at health centres (again excluding the cost of drugs and laboratory tests) for 191 countries. The second main source of data is the OHT, which provides default data on unit costs for drugs, personnel time and wages, and consumables (excluding drugs) [8]. Figure 1 illustrates the different sources of data used in calculating the cost per person/case per intervention visit (unit cost) in LiST.

### Calculating other-direct and indirect cost shares

Here we adapt the WHO CHOICE unit cost data to calculate the factor shares of the other-direct (excluding drugs, personnel and consumables) and indirect (capital/investment) costs, based on an analysis of costing data related to Voluntary Medical Male Circumcision (VMMC) [9]. Direct costs usually include initial personnel training, lab fees, gasoline, building rent, office supplies and promotional activities and publications. Indirect costs usually include support service costs like central support/management staff, international consultants, maintenance workers, "supervision," as well as drivers, insurance, utilities/telephone, publicity and other promotional activities, office furniture, other equipment such as autoclaves and typewriters, vehicle maintenance, other electronic maintenance, monitoring and evaluation. The disaggregated costing data used in this analysis are from facility-based data of VMMC in Kenya, Namibia, Uganda, South Africa, Zambia and Zimbabwe [10]. This analysis assumes that the labor utilization is comparable between VMMC and maternal and child health services; further details of the methodology, along with regression results, can be found in the Additional file. Note that the data are analyzed separately for hospitals and health centres.

As Table 1 shows, personnel costs account for the greatest share of costs for both hospitals and health centres, at 50 percent and 38 percent, respectively. The second largest contributor to costs here are indirect costs, followed by consumable costs and other-direct costs (i.e., excluding personnel, consumable and drug costs). There is a noticeable difference between consumables cost share at hospitals and health centres (21 percent versus 17 percent), however, the difference between hospitals and health centres for other-direct (7.5 percent versus 6.75 percent) and indirect (22 percent versus 23 percent) costs, are relatively small.

**Table 1:** Cost share of factors based on the translog cost function

Cost Share	Hospital	Health Center
**Personnel cost**	50%	39%
**Consumable cost**	21%	17%
**Direct cost**	7.5%	6.75%
**Indirect cost**	22%	23%

Source: These shares are generated from equation A3.3 in Additional file 1; they do not sum to one as the technical efficiency factor varies at different scales of operation. See Additional file 1 for further discussion.

### Calculating the other-direct and indirect costs from WHO CHOICE

The WHO CHOICE database provides the total unit cost for each facility visit by country and by type of facility, and assumes that a standard outpatient visit takes 20 minutes. We use Ghana as an example here: the cost per outpatient visit for health centres with no beds in Ghana is $1.29 (see tables in Additional file 1 for the cost of a visit at other types of facilities in Ghana). We calculate the other-direct and indirect costs by multiplying the facility level unit cost by the cost share factor from the analysis of VMMC cost data described above and in Additional file 1, resulting in $0.09 and $0.29 for health centres, respectively, for a total of $0.38 for the other-direct and indirect portions of unit cost. This same methodology is applied to calculate the other-direct and indirect costs for hospitals, using the relevant cost share factors from Table 1. These results will be utilized further below.

### Additional indirect and other-direct outreach cost

The methodology to calculate the indirect and other-direct costs for outreach visits is slightly different than the methodology for health centres and hospitals. We assume that additional outreach costs consist of incremental time and costs to a facility, calculated by the average transportation time for an outreach visit in one day divided by the number of cases seen in that day. For example, for an outreach visit that takes approximately 100 minutes for transportation to visit 20 patients, we assume that it takes five minutes of transportation time per case per visit. We then calculate this as a fraction of a standard (20 minute) visit, which equals $0.10 for health centres with no beds in Ghana, and add it to the initial other-direct and indirect costs from above ($0.38) to reach a total outreach cost in Ghana for other-direct and indirect costs of $0.48:

Equation 2: Other-direct/Indirect outreach cost ($0.48) =Other-direct/indirect visit cost ($0.38) + (5mins outreach time/20 min standard visit) x other-direct/indirect cost share factor x facility visit unit cost ($0.10)

### Cost of bundled visits

We focus here on a concrete example of the delivery of bundled interventions for antenatal interventions. To calculate the costs associated with delivering bundled interventions, we assume that there is a base intervention and all other interventions are incremental (recall that these assumptions are documented in [8]). Thus the incremental other-direct and indirect costs of each additional intervention are calculated as a fraction of a standard visit:

Equation 3: Other-direct incremental cost = (incremental minutes/20) x Other-direct cost share factor x facility visit unit cost.

Equation 4: Indirect incremental cost = (incremental minutes /20mins) x Indirect cost share factor x facility visit unit cost.

For example, the other-direct cost for an intervention that requires two incremental minutes provided at a health centre with no beds in Ghana would be (2/20) x 0.075 x $1.29 for a total of $.009. Using the same example, the indirect cost for an intervention that requires two incremental minutes provided at a health centre with no beds in Ghana would be (2/20) x 0.2 x $1.29 for a total of $0.03.

## Results

### Applying other-direct and indirect cost shares to build a comprehensive unit cost for LiST interventions

Recall the equation for calculating the unit cost of a LiST intervention:

Equation 1: Unit Cost = Personnel + Drugs + Consumables + Other direct + Indirect

Given the cost shares for both other-direct and indirect costs, in the previous section we calculated the proportion of these costs associated with the WHO CHOICE health centre visit costs. We combine these costs with the default costs for personnel, drugs and consumables available from OHT to calculate the unit cost for the interventions being examined. We then calculate potential cost savings associated with delivering interventions in a bundled way versus delivering the intervention alone.

Below is a step by step example of the unit cost calculation using ANC visits as the base intervention.

### Personnel, drug and consumable costs from One Health Model (OHT)

The personnel, drug and consumable costs per visit for each of the six interventions are taken from the OHT for Ghana [8], and are shown in the first set of columns in Table 2. As an example, the cost for a basic ANC visit consists of $0.68 for drugs, $0.28 for personnel, and $0.25 for consumables, for a total for the three components of $1.20. Note that the ANC example assumes that services are being provided by nurses (in the facility as well as in outreach) who are paid at an approximate rate of $0.01 per minute. The second set of columns displays the annual cost based on the WHO-recommended number of visits per pregnancy per year. Note that the default costs from OHT for the other five interventions to be examined are also displayed in Table 2.

**Table 2:** Drugs, personnel and consumable costs for ANC visits

	Cost per visit				Annual cost				
	
	Drug costs	Personnel costs	Consumable costs	Combined personnel, drugs and consumable cost	Recommended number of visits per pregnancy/year	Drug costs	Personnel costs	Consumable costs	Combined personnel, drugs and consumable cost
**Basic ANC visit**	$0.68	$0.28	$0.25	$1.21	4	$2.72	$1.11	$0.98	$4.81
**Syphilis detection and treatment**	$0.26	$0.28	$0.11	$0.65	1	$0.26	$0.28	$0.11	$0.65
**Tetanus toxoid**	$0.09	$0.28	$0.05	$0.42	2	$0.17	$0.56	$0.10	$0.83
**Balanced energy supplementation**	$6.00	$0.28	$0.00	$6.28	4	$24.00	$1.11	$0.00	$25.11
**Multiple micronutrient supplementation**	$0.02	$0.28	$0.00	$0.30	4	$0.07	$1.11	$0.00	$1.18
**Pregnant women protected via IPT**	$0.01	$0.28	$0.00	$0.29	2	$0.02	$0.56	$0.00	$0.58

Source: Assumptions are based on expert opinion and are documented in WHO OHT [Futures Institute, [8]]

### Calculating Other-direct and Indirect Costs from WHO CHOICE

Recall that, using the estimated cost share from the analysis of VMMC data, we calculated that other-direct costs (direct costs less personnel, drug and consumable costs) and indirect costs account for approximately 6.7% and 23% of the WHO CHOICE unit cost of visits at health centres, respectively (see Table 1, above). As described above, each ANC visit is estimated to take 20 minutes and other-direct and indirect costs per visit are calculated to be $0.09 and $0.29, respectively. The annual other-direct and indirect costs for interventions with four recommended visits per year (assuming four visits in a year) are thus $0.37 and $1.17, while the other-direct and indirect costs for interventions with two recommended visits are $0.18 and $0.59, respectively (see Table 3).

**Table 3:** Other-direct (excluding personnel, consumable and drug costs) and indirect costs per visit and annually for ANC interventions delivered at a health centre in Ghana

	Per visit cost		Annual cost	
	**Other-Direct cost**	**Indirect cost**	**Other-Direct cost**	**Indirect cost**

**Basic ANC visit**	$0.09	$0.29	$0.37	$1.17
**Syphilis detection and treatment**	$0.09	$ 0.29	$0.18	$ 0.59
**Tetanus toxoid**	$0.09	$ 0.29	$0.18	$ 0.59
**Balanced energy supplementation**	$0.09	$ 0.29	$0.37	$ 1.17
**Multiple micronutrient supplementation**	$0.09	$ 0.29	$0.37	$ 1.17
**Pregnant women protected via IPT**	$0.09	$ 0.29	$0.18	$ 0.59

Source: Table 1 and authors’ calculations as applied to Ghana data.

### Calculating costs of bundling interventions

We then apply the other-direct and indirect cost shares to the six kinds of ANC visits- basic ANC, tetanus toxoid, syphilis detection and treatment, balanced energy supplementation, multiple micronutrient supplementation and malaria for pregnant women via Intermittent Preventive Treatment (IPT). Recall that the incremental other-direct and indirect costs are calculated based on the incremental number of minutes estimated to deliver the intervention along with the base intervention, the basic ANC visit [8].

Table 4 shows the other-direct and indirect base and incremental time (i.e. number of minutes when added to the basic ANC visit), costs per visit and annual costs (assuming all WHO-recommended visits are completed). Because the base intervention, a basic ANC visit, takes 20 minutes, the other-direct and indirect costs per visit as well as the annual costs remain the same as in Table 3 above. The time associated with delivering the other five interventions, however, decreases, thereby decreasing the associated other-direct and indirect costs. The other-direct and indirect costs of interventions that have a five minute incremental visit - syphilis detection and treatment, balanced energy supplementation and multiple micronutrient supplementation - are 75 percent less when bundled with the basic ANC visit than when the same interventions are offered alone. Similarly, interventions that have a two minute incremental visit - tetanus toxoid and IPT for pregnant women - have other-direct and indirect costs that are 90 percent less when delivered with the basic ANC visit.

**Table 4:** Incremental other-direct (excluding personnel, consumable and drug costs) and indirect visit time, per visit and annual costs

		Per visit cost		Annual cost	
	**Incremental visit time in minutes**	**Other-Direct cost**	**Indirect cost**	**Other-Direct cost**	**Indirect cost**

**Basic ANC visit**	20 (base visit)	$0.09	$0.29	$0.36	$1.17
**Syphilis detection and treatment**	5	$0.023	$0.07	$0.046	$0.14
**Tetanus toxoid**	2	$0.01	$0.029	$0.018	$0.059
**Balanced energy supplementation**	5	$0.023	$0.07	$0.092	$0.29
**Multiple micronutrient supplementation**	5	$0.023	$0.07	$0.092	$0.29
**Pregnant women protected via IPT**	2	$0.01	$0.029	$0.018	$0.059

Source: WHO OHT [8] and authors’ calculations

### Comparing unit costs of bundling services versus delivering separately

Finally, we calculate a total cost for each intervention by aggregating the different cost components in Table 4, and compare the overall costs of interventions when delivered separately versus bundled with the basic ANC visit. We present aggregated costs in three major categories (see Table 5):

1) the unit cost of an ANC visit delivered at a health centre;

2) the unit cost per pregnancy to provide the recommended number of visits to each case assuming the different types of ANC visits are provided separately; and

3) the unit cost per case, assuming the different types of ANC visits are bundled together.

**Table 5:** Cost comparison of ANC visit delivered at a health centre with no beds in Ghana

	Unit cost per visit	Annual unit cost of intervention (delivered separately)	Annual unit cost of intervention (delivered as a bundle)
Basic ANC visit	$4.53	$9.15	$9.15
Syphilis detection and treatment	$1.07	$2.16	$0.86
Tetanus toxoid	$0.99	$2.00	$0.85
Balanced energy supplementation	$24.78	$27.14	$24.780
Multiple micronutrient supplementation	$24.78	$27.14	$24.78
Pregnant women protected via IPT	$0.84	$1.70	$0.35

**Total cost**		$69.29	$60.77

**Cost savings from bundling interventions**			$8.52 (12.2%)

Source: Authors’ calculations

The total cost of delivering all six interventions separately is $69.76, while when the six interventions are delivered as a bundle the cost drops to $61.18, a cost savings of $8.58, or 12.2 percent. Note that these savings are based on very conservative estimates of cost-savings, related only to lower amounts of personnel time and a related reduction in other-direct and indirect costs at the facility level. In addition, the savings are invariant with respect to scaling-up coverage of interventions; that is, the reduction in cost will be 12.2 percent if coverage increases by five percentage points or fifty.

Note that in the LiST model, costing results can be displayed in many different ways. In addition to total intervention costs, sub-categories of costs can also be displayed including drugs, supplies, or a combination of drugs and supplies costs; labor costs; other direct costs (excluding drug, supplies and labor costs); and indirect costs. Costs can also be displayed by delivery channel (community, outreach, clinic, hospital), currency (as defined by the user, usually local currency and US dollar), and by either total or incremental cost.

### Limitations of the analysis

This analysis has certain limitations. Due to data availability constraints, the cost share factor analysis is based on VMMC unit cost data, and thus assumes that the cost share factors are the same for maternal and child health services. In addition, the cost of the set of commodities used in implementing VMMC may not be the same as those used in maternal and child health visits, which would affect the estimated cost shares. Finally, although the assumptions used in calculating incremental costs for bundling services are from WHO and other experts, and thus based on their field experience, it will be important to further validate these assumptions empirically.

## Conclusions

In order to make effective decisions on how to allocate limited resources to maternal and child health services, program planners and policymakers must be armed with both impact and cost analyses. We develop a method for costing using cost share regression analysis drawing on existing unit cost data and the literature on cost analysis to develop a comprehensive cost estimate of maternal and child health intervention critical to the decision making process. We then combine the resulting calculated unit costs with estimates of incremental time to determine whether cost savings associated with bundling or combining the delivery of interventions in one outpatient visit exist. Using ANC visits as an example, we find that delivering additional services at the basic ANC visit results in a cost savings of 12.2 percent. With this analysis, the LiST tool can now provide the cost of delivering interventions alone by combining personnel, consumable and drug costs from OHT with the indirect and other-direct costs from the respective proportions of the WHO CHOICE unit cost. Future changes to LiST costing will provide users with the ability to bundle interventions and compare estimated cost-savings from bundling interventions. In addition, we plan to apply this method to other modules in Spectrum, beginning with the FamPlan module.

The costing method proposed by this paper is one of many steps towards developing estimates of the comprehensive cost of delivering maternal and child health services in the context of a flexible health policy and planning tool. The methodology will be particularly useful for calculating the costs of scaling-up programs when they will be delivered in a bundled fashion. Combining the savings of bundling interventions with the added impact of delivering the additional interventions offers policy makers and program planners in resource limited settings strategies for cost-effective expansion of maternal and child health services.

## List of abbreviations used

ANC: AnteNatal Care; ART: AntiRetroviral Therapy; CHOICE: CHOosing Interventions that are Cost-Effective; HIV: Human Immunodeficiency Virus; IPT: Intermittent Presumptive Treatment; LiST: Lives Saved Tool; OHT: OneHealth Tool; STI: Sexually Transmitted Infections; VMMC: Voluntary Medical Male Circumcision; WHO: World Health Organization.

## Competing interests

The authors declare that they have no competing interests.

## Authors' contributions

Both AA and LB made substantial contributions to conception and design, acquisition of data, and analysis and interpretation of data; have been involved in drafting the manuscript or revising it critically for important intellectual content; and have given final approval of the version to be published.

## Supplementary Material

Additional file 1 Detailed methods Click here for file

## Declarations

The publication costs for this supplement were funded by a grant from the Bill & Melinda Gates Foundation to the US Fund for UNICEF (grant 43386 to "Promote evidence-based decision making in designing maternal, neonatal, and child health interventions in low- and middle-income countries”). The Supplement Editor is the principal investigator and lead in the development of the Lives Saved Tool (LiST), supported by grant 43386. He declares that he has no competing interests.

This article has been published as part of *BMC Public Health* Volume 13 Supplement 3, 2013: The Lives Saved Tool in 2013: new capabilities and applications. The full contents of the supplement are available online at http://www.biomedcentral.com/bmcpublichealth/supplements/13/S3.
