# Supplementary material for: Estimating the cost-savings associated with bundling maternal and child health interventions: a proposed methodology
Source: BMC Public Health. 2013 Sep 17;13(Suppl 3):S27. doi: 10.1186/1471-2458-13-S3-S27 (PMC3847650; doi:10.1186/1471-2458-13-S3-S27)
Supplement: Additional file 1 — Detailed methods [file 1471-2458-13-S3-S27-S1.docx]

# Additional files

### Additional file 1 – Annex A

Following Berndt and Wood [1] and Diewert [2], we assume a unit cost function exists that reflects a well-behaved production function consisting of four factors of production:

Equation A1: U = U(P, C, O, I)

where:

- U = unit cost,
- P = personnel unit cost,
- C = consumables unit cost,
- O = other-direct unit cost (variable/recurrent costs excluding personnel and consumable cost), and
- I = indirect unit cost (capital/investment cost).

Applying Shepherd’s Lemma, and assuming that the cost function is also well-behaved, then

Equation A2: x_i_ (Q; p) = δU (Q; p) ; i = P, C, O, I

δp_i_

where x_i_ (Q; p) is the cost minimizing demand for input i needed to produce output Q≥0 given factor prices p»0.

Again following the literature we use a generalized approach to calculate the unit cost function through estimating a translog (transcendental logarithmic) cost function [Berndt and Wood, 1; Diewert, 2; Kumbhakar, 3]. The four-input unit cost function from above can be written in natural logarithmic terms as follows, limiting the cross-terms to first-derivatives only:

Equation A2: ln U = ln α_0_ + α_P_ ln P + α_C_ ln C + α_O_ ln O + α_I_ ln I + β_PP_ ln P^2^

+ β_CC_ ln C^2^ + β_OO_ ln O^2^ + β_II_ ln I^2^ + β_PC_ ln P ln C + β_PO_ ln P ln O

+ β_PI_ ln P ln I + β_CO_ ln C ln O + β_CI_ ln C ln I + β_OI_ ln O ln I

where:

- U is the unit cost for VMMC,
- P, C, O and I are the personnel, consumables, other direct (variable or recurrent costs excluding personnel and consumables cost) and indirect (capital or investment) unit costs for a VMMC patient, respectively,
- α_0_ represents the intercept while α_P_, α_C_, α_O_ and α_I_ are the coefficients for the first-order terms,
- β_PP_, β_CC_, β_OO_ and β_II_ are the coefficients for the second-order terms, and
- β_PC_, β_PO_, β_PI_, β_CO_, β_CI_ and β_OI_ are the coefficients for the first-order cross terms.

Differentiating Equation A2 to derive factor shares results in the following equations:

Equation A3: Unit cost share of Personnel costs

δln U/ln P = α_P_ + 2 β_PP_ ln P + β_PC_ ln C + β_PO_ ln O + β_PI_ ln I

Equation A4: Unit cost share of Consumables costs

δln U/ln C = α_C_ + 2 β_CC_ ln C + β_CP_ ln P + β_CO_ ln O + β_CI_ ln I

Equation A5: Unit cost share of Other-direct costs

δ ln U/ln O = α_O_ + 2 β_OO_ ln O + β_OP_ ln P + β_OC_ LN CO + β_OI_ ln I

Equation A6: Unit cost share of Indirect costs

δ ln U/ln I = α_I_ + 2 β_II_ ln I + β_IP_ ln P + β_IC_ ln C + β_IO_ ln O

The factor shares are constants that are determined by available technology [Deaton and Muellbauer, 4] and represent the responsiveness of unit cost to changes in the levels of personnel, consumables, other-direct and indirect cost such that:

- If α_P_  + α_C_ + α_O_ + α_I_ = 1, then the function has constant returns to scale,
- If α_P_  + α_C_ + α_O_ + α_I_ < 1, then the function has decreasing returns to scale, and
- If α_P_  + α_C_ + α_O_ + α_I_ > 1, then the function has increasing returns to scale.

The translog cost function was estimated using facility-level data to obtain estimates for a four-input unit cost symmetric function. In order to obtain the cost share for personnel, consumable, direct and indirect costs, the translog function was differentiated with respect to each factor input as delineated in equations A3-A6 above evaluating the variables at their means (see Table A1).

Table A1: Descriptive statistics for regression variables: mean and standard deviations

|  | Mean | Standard deviation |
| --- | --- | --- |
| Hospital unit cost | $ 48.68 | $ 26.59 |
| Personnel | $ 20.01 | $ 18.81 |
| Consumables | $ 9.95 | $ 8.57 |
| Other-direct | $ 1.92 | $ 2.76 |
| Indirect | $ 12.70 | $ 12.51 |
|  |  |  |
| Health centre unit cost | $ 37.79 | $ 19.06 |
| Personnel | $ 14.60 | $ 13.04 |
| Consumables | $ 8.33 | $ 4.79 |
| Other-direct | $ 2.16 | $ 3.15 |
| Indirect | $ 12.70 | $ 12.51 |

## Regression results from estimation of translog function: hospitals and health centres

| Multiple R | 0.994716 |  |  | *df* | *SS* | *MS* | *F* | *Significance F* |
| --- | --- | --- | --- | --- | --- | --- | --- | --- |
| R Square | 0.989461 |  | Regression | 14 | 23.54851 | 1.682036 | 268.2364 | 6.20E-35 |
| Adjusted R Square | 0.985772 |  | Residual | 40 | 0.250829 | 0.006271 |  |  |
| Standard Error | 0.079188 |  | Total | 54 | 23.79934 |  |  |  |
| Observations | 55 |  |  |  |  |  |  |  |
|  |  |  |  |  |  |  |  |  |
|  | *Coefficients* | *Standard Error* | *t Stat* | *P-value* | *Lower 95%* | *Upper 95%* | *Lower 95.0%* | *Upper 95.0%* |
| α_0_ | 1.581804 | 0.182635 | 8.661013 | 1.03E-10 | 1.212685 | 1.950923 | 1.212685 | 1.950923 |
| α_1_ | 0.059194 | 0.085004 | 0.696368 | 0.490226 | -0.11261 | 0.230994 | -0.11261 | 0.230994 |
| α_2_ | 0.321096 | 0.138269 | 2.322256 | 0.025393 | 0.041644 | 0.600548 | 0.041644 | 0.600548 |
| α_3_ | 0.129391 | 0.028361 | 4.562327 | 4.72E-05 | 0.072072 | 0.186711 | 0.072072 | 0.186711 |
| α_4_ | 0.396705 | 0.039876 | 9.948479 | 2.24E-12 | 0.316113 | 0.477297 | 0.316113 | 0.477297 |
| α_5_ | 0.092096 | 0.015461 | 5.956509 | 5.44E-07 | 0.060847 | 0.123345 | 0.060847 | 0.123345 |
| α_6_ | 0.059097 | 0.027059 | 2.183987 | 0.034886 | 0.004408 | 0.113785 | 0.004408 | 0.113785 |
| α_7_ | 0.006052 | 0.001858 | 3.257351 | 0.002296 | 0.002297 | 0.009808 | 0.002297 | 0.009808 |
| α_8_ | 0.047146 | 0.005592 | 8.430744 | 2.08E-10 | 0.035844 | 0.058449 | 0.035844 | 0.058449 |
| α_9_ | -0.02271 | 0.025819 | -0.8795 | 0.384384 | -0.07489 | 0.029475 | -0.07489 | 0.029475 |
| α_10_ | 0.007367 | 0.005333 | 1.381472 | 0.174802 | -0.00341 | 0.018145 | -0.00341 | 0.018145 |
| α_11_ | -0.0273 | 0.019012 | -1.43574 | 0.158851 | -0.06572 | 0.011128 | -0.06572 | 0.011128 |
| α_12_ | -0.01352 | 0.008645 | -1.56387 | 0.125726 | -0.03099 | 0.003953 | -0.03099 | 0.003953 |
| α_13_ | -0.13159 | 0.026255 | -5.01204 | 1.14E-05 | -0.18466 | -0.07853 | -0.18466 | -0.07853 |
| α_14_ | -0.02246 | 0.006924 | -3.24341 | 0.002387 | -0.03645 | -0.00846 | -0.03645 | -0.00846 |

Table A2: Summary Output for Hospitals

Table A3: Summary Output for Health Centres

| Multiple R | 0.995723 |  |  | *df* | *SS* | *MS* | *F* | *Significance F* |
| --- | --- | --- | --- | --- | --- | --- | --- | --- |
| R Square | 0.991464 |  | Regression | 14 | 10.5211 | 0.751507 | 157.6246 | 1.35E-16 |
| Adjusted R Square | 0.985173 |  | Residual | 19 | 0.090586 | 0.004768 |  |  |
| Standard Error | 0.069049 |  | Total | 33 | 10.61168 |  |  |  |
| Observations | 34 |  |  |  |  |  |  |  |
|  |  |  |  |  |  |  |  |  |
|  | *Coefficients* | *Standard Error* | *t Stat* | *P-value* | *Lower 95%* | *Upper 95%* | *Lower 95.0%* | *Upper 95.0%* |
| α_0_ | 2.130559 | 0.215738 | 9.87566 | 6.44E-09 | 1.679014 | 2.582105 | 1.679014 | 2.582105 |
| α_1_ | 0.00855 | 0.095512 | 0.089513 | 0.929611 | -0.19136 | 0.208459 | -0.19136 | 0.208459 |
| α_2_ | -0.41843 | 0.235219 | -1.77889 | 0.091262 | -0.91075 | 0.07389 | -0.91075 | 0.07389 |
| α_3_ | 0.251763 | 0.040136 | 6.272773 | 5.06E-06 | 0.167758 | 0.335768 | 0.167758 | 0.335768 |
| α_4_ | 0.631621 | 0.097327 | 6.48965 | 3.22E-06 | 0.427912 | 0.835329 | 0.427912 | 0.835329 |
| α_5_ | 0.09667 | 0.015844 | 6.101484 | 7.24E-06 | 0.063509 | 0.129831 | 0.063509 | 0.129831 |
| α_6_ | 0.273049 | 0.084648 | 3.2257 | 0.00445 | 0.095879 | 0.450219 | 0.095879 | 0.450219 |
| α_7_ | 0.005635 | 0.002302 | 2.44732 | 0.024285 | 0.000816 | 0.010454 | 0.000816 | 0.010454 |
| α_8_ | 0.077496 | 0.02253 | 3.439717 | 0.002746 | 0.03034 | 0.124651 | 0.03034 | 0.124651 |
| α_9_ | 0.040326 | 0.027226 | 1.481165 | 0.154957 | -0.01666 | 0.09731 | -0.01666 | 0.09731 |
| α_10_ | -0.00571 | 0.004914 | -1.16236 | 0.259482 | -0.016 | 0.004573 | -0.016 | 0.004573 |
| α_11_ | -0.08871 | 0.020728 | -4.27977 | 0.000405 | -0.13209 | -0.04533 | -0.13209 | -0.04533 |
| α_12_ | -0.04865 | 0.020274 | -2.39949 | 0.026834 | -0.09108 | -0.00621 | -0.09108 | -0.00621 |
| α_13_ | -0.25212 | 0.069362 | -3.63484 | 0.001763 | -0.3973 | -0.10694 | -0.3973 | -0.10694 |
| α_14_ | -0.02928 | 0.011501 | -2.54578 | 0.019735 | -0.05335 | -0.00521 | -0.05335 | -0.00521 |

Table A4: Cost per outpatient visit in Ghana (USD) from WHO CHOICE

|  |  | Health Centre no beds | Health Centre with beds | Primary-level hospital | Secondary-level hospital |
| --- | --- | --- | --- | --- | --- |
| Cost per outpatient visit US$ | | $ 1.29 | $ 1.59 | $ 1.81 | $ 1.89 |

Table A5: Direct and Indirect Costs per patient by facility type (outreach costs are calculated by adding the cost of an additional 5 minutes per case per visit)

|  |  | Health Centre no beds | Health Centre with beds | Primary-level hospital | Secondary-level hospital |
| --- | --- | --- | --- | --- | --- |
| Direct Cost | | $0.09 | $0.11 | $0.14 | $0.14 |
| Direct Cost outreach | | $0.11 | $0.13 | $0.17 | $0.18 |
| Indirect Cost | | $0.30 | $0.37 | $0.40 | $0.42 |
| Indirect Cost outreach cost | | $0.37 | $0.46 | $0.50 | $0.52 |

Table A6: Direct and indirect costs of basic ANC visit per case by facility type

|  |  | Health Centre no beds | Health Centre with beds | Primary-level hospital | Secondary-level hospital |
| --- | --- | --- | --- | --- | --- |
| Direct Cost | | $0.09 | $0.11 | $0.14 | $0.14 |
| Direct Cost outreach | | $0.11 | $0.13 | $0.17 | $0.18 |
| Indirect Cost | | $0.30 | $0.37 | $0.40 | $0.42 |
| Indirect Cost outreach | | $0.37 | $0.46 | $0.50 | $0.52 |

Table A7: Annual other-direct and indirect costs of basic ANC visit by facility type (recommended number of visits = 4)

|  |  | Health Centre no beds | Health Centre with beds | Primary-level hospital | Secondary-level hospital |
| --- | --- | --- | --- | --- | --- |
| Other-Direct Cost | | $0.35 | $0.43 | $0.54 | $0.57 |
| Other-Direct Cost (outreach) cost) | | $0.44 | $0.54 | $0.68 | $0.71 |
| Indirect Cost | | $1.19 | $1.46 | $1.59 | $1.66 |
| Indirect Cost (outreach cost) | | $1.48 | $1.83 | $1.99 | $2.08 |

Table A8: Other-Direct and indirect costs per visit per case for syphilis screening or balanced energy or multiple micronutrient supplementation visit by facility type (incremental cost for adding to basic ANC in parentheses)

|  |  | Health Centre  no beds | Health Centre with beds | Primary-level hospital | Secondary-level hospital |
| --- | --- | --- | --- | --- | --- |
| Other-Direct Cost | | $0.09 ($0.022) | $0.11 ($0.027) | $0.14 ($0.034) | $0.14 ($0.035) |
| Other-Direct Cost outreach | | $0.11 ($0.022) | $0.13 ($0.027) | $0.17 ($0.034) | $0.18 ($0.035) |
| Indirect Cost | | $ 0.30 ($0.07) | $0.37 ($0.09) | $0.40($0.10) | $ 0.42 (0.10) |
| Indirect Cost outreach | | $0.37 ($0.07) | $ 0.46 ($0.09) | $0.50 ($0.10) | $0.52 ($0.10) |

Table A9: Annual other-direct and indirect cost per case for syphilis screening visit by facility type (incremental cost for adding to basic ANC in parentheses, recommended number of visits = 2)

|  |  | Health Centre  no beds | Health Centre with beds | Primary-level hospital | Secondary-level hospital |
| --- | --- | --- | --- | --- | --- |
| Other-Direct Cost | | $0.18 ($0.044) | $0.22 ($0.054) | $0.28 ($0.068) | $0.28 ($0.07) |
| Other-Direct Cost (outreach cost) | | $0.22 ($0.044) | $0.26 ($0.054) | $0.34 ($0.068) | $0.36 ($0.07) |
| Indirect Cost | | $ 0.60 ($0.14) | $0.74 ($0.18) | $0.80 ($0.20) | $ 0.84 (0.20) |
| Indirect Cost (outreach cost) | | $0.74 ($0.14) | $ 0.98 ($0.18) | $1.00 ($0.20) | $1.04 ($0.20) |

Table A10: Annual other-direct and indirect cost per case for balanced energy or multiple micronutrient supplement visit by facility type (incremental cost for adding to basic ANC in parentheses, recommended number of visits = 4)

|  |  | Health Centre  no beds | Health Centre with beds | Primary-level hospital | Secondary-level hospital |
| --- | --- | --- | --- | --- | --- |
| Other-Direct Cost | | $0.36 ($0.088) | $0.44 ($0.108) | $0.48 ($0.136) | $0.48 ($0.14) |
| Other-Direct Cost (outreach cost) | | $0.44 ($0.088) | $0.56 ($0.108) | $0.60 ($0.136) | $0.60 ($0.14) |
| Indirect Cost | | $ 1.28 ($0.28) | $1.56 ($0.36) | $2.24($0.40) | $2.36 ($0.40) |
| Indirect Cost (outreach cost) | | $1.60 ($0.28) | $1.96 ($0.36) | $2.80 ($0.40) | $ 2.92 ($0.40) |

Table A11: Other-direct and indirect costs for per case for tetanus toxoid/ pregnant women protected via IPT (malaria) visit by facility type (incremental cost for adding to basic ANC in parentheses)

|  |  | Health Centre  no beds | Health Centre - with bed | Primary-level hospital | Secondary-level hospital |
| --- | --- | --- | --- | --- | --- |
| Other-Direct Cost | | $0.09 ($0.009) | $0.11 ($0.01) | $0.11 ($0.011) | $0.11 ($0.011) |
| Other-Direct Cost (outreach cost) | | $0.11 ($0.009) | $0.13 ($0.01) | $0.17 ($0.011) | $0.18 ($0.011) |
| Indirect Cost | | $ 0.30($0.03) | $0.37 ($0.037) | $0.40 ($0.04) | $ 0.42 (0.04) |
| Indirect Cost (outreach cost) | | $0.37 ($0.03) | $ 0.46 ($0.046) | $0.50 ($0.05) | $0.52 ($0.05) |

Table A12: Annual other-direct and indirect costs per case for tetanus toxoid/ pregnant women protected via IPT (malaria) visit by facility type (incremental cost for adding to basic ANC in parentheses, recommended number of visits = 2)

|  |  | Health Centre  no beds | Health Centre with beds | Primary-level hospital | Secondary-level hospital |
| --- | --- | --- | --- | --- | --- |
| Other-Direct Cost | | $0.18($0.018) | $0.23 ($0.02) | $0.24 ($0.022) | $0.25 ($0.022) |
| Other-Direct Cost (outreach cost) | | $0.22 ($0.018) | $0.28 ($0.02) | $0.29 ($0.022) | $0.31 ($0.022) |
| Indirect Cost | | $ 0.30($0.06) | $0.74 ($0.074) | $0.80 ($0.08) | $0.84 ($ 0.08) |
| Indirect Cost (outreach cost) | | $0.74 ($0.06) | $ 0.98 ($0.074) | $1.00 ($0.08) | $1.04($ 0.08) |

**Unit Cost per ANC visit**

Table A13: Basic ANC visit unit cost by facility type (Basic ANC+ other-direct ANC + indirect ANC: for facility or outreach level). The difference in cost between the facility types is the change in other-direct and indirect costs by facility level, given that the amount of time and the number of drugs and supplies remains the same across facilities.

|  |  | Health Centre no beds | Health Centre with beds | Primary-level hospital | Secondary-level hospital |
| --- | --- | --- | --- | --- | --- |
| Facility visit costs | | $4.57 | $4.66 | $4.62 | $4.65 |
| Outreach visit costs | | $4.77 | $4.88 | $4.83 | $4.87 |

Table A14: Syphilis screening by facility type (syphilis screening + other-direct syphilis + indirect syphilis: for facility or outreach level)

|  |  | Health Centre no beds | Health Centre with beds | Primary-level hospital | Secondary-level hospital |
| --- | --- | --- | --- | --- | --- |
| Facility visit costs | | $1.07 | $1.17 | $1.22 | $1.25 |
| Outreach visit costs | | $1.27 | $1.38 | $1.46 | $1.50 |

Table A15: Tetanus toxoid by facility type

|  |  | Health Centre no beds | Health Centre with beds | Primary-level hospital | Secondary-level hospital |
| --- | --- | --- | --- | --- | --- |
| Facility visit costs | | $0.99 | $1.17 | $1.22 | $1.25 |
| Outreach visit costs | | $1.19 | $1.38 | $1.46 | $1.50 |

Table A16: Pregnant women protected via IPT (Malaria) visit by facility type

|  |  | Health Centre no beds | Health Centre with beds | Primary-level hospital | Secondary-level hospital |
| --- | --- | --- | --- | --- | --- |
| Facility visit costs | | $0.84 | $1.17 | $1.22 | $1.25 |
| Outreach visit costs | | $1.04 | $1.38 | $1.46 | $1.50 |

Table A17: Balanced energy supplement by facility type

|  |  | Health Centre no beds | Health Centre with beds | Primary-level hospital | Secondary-level hospital |
| --- | --- | --- | --- | --- | --- |
| Facility visit costs | | $24.78 | $24.88 | $24.93 | $24.96 |
| Outreach visit costs | | $24.98 | $25.09 | $25.17 | $25.21 |

Table A18: Multiple micronutrient supplement by facility type

|  |  | Health Centre no beds | Health Centre with beds | Primary-level hospital | Secondary-level hospital |
| --- | --- | --- | --- | --- | --- |
| Facility visit costs | | $24.78 | $24.88 | $24.93 | $24.96 |
| Outreach visit costs | | $24.98 | $25.09 | $25.17 | $25.21 |

**Annual unit cost per case (assuming interventions are delivered separately)** (annual cost per case for type of intervention + annual other-direct cost +annual indirect cost: for facility or outreach)

Table A19: Basic ANC visit unit cost by facility type

|  |  | Health Centre no beds | Health Centre with beds | Primary-level hospital | Secondary-level hospital |
| --- | --- | --- | --- | --- | --- |
| Facility visit costs | | $9.15 | $9.50 | $9.35 | $9.44 |
| Outreach visit costs | | $9.93 | $10.38 | $10.16 | $10.32 |

Table A20: Syphilis screening by facility type

|  |  | Health Centre no beds | Health Centre with beds | Primary-level hospital | Secondary-level hospital |
| --- | --- | --- | --- | --- | --- |
| Facility visit costs | | $2.16 | $2.35 | $2.45 | $2.50 |
| Outreach visit costs | | $2.54 | $2.76 | $2.91 | $2.98 |

Table A21: Tetanus toxoid by facility type

|  |  | Health Centre  no beds | Health Centre with beds | Primary-level hospital | Secondary-level hospital |
| --- | --- | --- | --- | --- | --- |
| Facility visit costs | | $2.00 | $2.19 | $2.29 | $2.34 |
| Outreach visit costs | | $2.38 | $2.60 | $2.75 | $2.82 |

Table A22: Pregnant women protected via IPT (Malaria) visit by facility type

|  |  | Health Centre  no beds | Health Centre with beds | Primary-level hospital | Secondary-level hospital |
| --- | --- | --- | --- | --- | --- |
| Facility visit costs | | $1.70 | $1.89 | $1.99 | $2.04 |
| Outreach visit costs | | $2.08 | $2.30 | $2.45 | $2.52 |

Table A23: Balanced energy supplement by facility type

|  |  | Health Centre  no beds | Health Centre- with beds | Primary-level hospital | Secondary-level hospital |
| --- | --- | --- | --- | --- | --- |
| Facility visit costs | | $27.15 | $27.52 | $27.74 | $27.83 |
| Outreach visit costs | | $27.92 | $28.37 | $28.67 | $28.82 |

Table A24: Multiple micronutrient supplement by facility type

|  |  | Health Centre no beds | Health Centre with beds | Primary-level hospital | Secondary-level hospital |
| --- | --- | --- | --- | --- | --- |
| Facility visit costs | | $27.15 | $27.52 | $27.74 | $27.83 |
| Outreach visit costs | | $27.92 | $28.37 | $28.67 | $28.82 |

Table A25: Annual cost of ANC visits when delivered separately by facility type

|  |  | Health Centre  no beds | Health Centre with beds | Primary-level hospital | Secondary-level hospital |
| --- | --- | --- | --- | --- | --- |
| Facility visit costs | | $69.29 | $70.96 | $71.54 | $71.97 |
| Outreach visit costs | | $72.76 | $74.77 | $75.62 | $76.28 |

**Annual unit cost assuming interventions are bundled**

Table A26: Basic ANC visit (fixed cost/fixed intervention) by facility type (incremental cost per case per year + incremental other-direct cost + incremental indirect cost: for facility or outreach)

|  |  | Health Centre  no beds | Health Centre - with bed | Primary-level hospital | Secondary-level hospital |
| --- | --- | --- | --- | --- | --- |
| Facility visit costs | | $9.15 | $9.50 | $9.35 | $9.44 |
| Outreach visit costs | | $9.93 | $10.38 | $10.16 | $10.32 |

Table A27: Syphilis screening (incremental cost) by facility type (annual incremental cost per case per year for syphilis screening + annual incremental syphilis screening other-direct cost + annual incremental syphilis screening indirect cost: for facility and outreach)

|  |  | Health Centre  no beds | Health Centre with beds | Primary-level hospital | Secondary-level hospital |
| --- | --- | --- | --- | --- | --- |
| Facility and Outreach | | $0.98 | $1.02 | $1.08 | $1.09 |

Table A28: Tetanus toxoid by facility type

|  |  | Health Centre  no beds | Health Centre with beds | Primary-level hospital | Secondary-level hospital |
| --- | --- | --- | --- | --- | --- |
| Facility and Outreach | | $0.90 | $0.92 | $1.16 | $1.16 |

Table A29: Pregnant women protected via IPT (malaria) visit by facility type

|  |  | Health Centre  no beds | Health Centre with beds | Primary-level hospital | Secondary-level hospital |
| --- | --- | --- | --- | --- | --- |
| Facility and Outreach | | $0.40 | $0.42 | $0.66 | $0.66 |

Table A30: Balanced energy supplement by facility type

|  |  | Health Centre  no beds | Health Centre with beds | Primary-level hospital | Secondary-level hospital |
| --- | --- | --- | --- | --- | --- |
| Facility and Outreach | | $24.80 | $24.90 | $25.04 | $25.07 |

Table A31: Multiple micronutrient supplement by facility type

|  |  | Health Centre  no beds | Health Centre with beds | Primary-level hospital | Secondary-level hospital |
| --- | --- | --- | --- | --- | --- |
| Facility and Outreach | | $24.80 | $24.90 | $25.04 | $25.07 |

Table A32: Annual unit cost of bundling ANC visits by facility type

|  |  | Health Centre  no beds | Health Centre with beds | Primary-level hospital | Secondary-level hospital |
| --- | --- | --- | --- | --- | --- |
| Facility visit costs | | $61.03 | $61.67 | $62.33 | $62.51 |
| Outreach visit costs | | $61.81 | $62.54 | $63.14 | $63.38 |

Table A33: Cost savings by facility type

|  |  | Health Centre  no beds | Health Centre with beds | Primary-level hospital | Secondary-level hospital |
| --- | --- | --- | --- | --- | --- |
| Facility visit costs | | $8.26 | $9.30 | $9.21 | $9.46 |
| Outreach visit costs | | $10.95 | $12.23 | $12.48 | $12.90 |

Table A34: Cost savings in percentage terms by facility type

|  |  | Health Centre  no beds | Health Centre with beds | Primary-level hospital | Secondary-level hospital | Health Centre  no beds |
| --- | --- | --- | --- | --- | --- | --- |
| Facility visit costs | | 12.2% | 13.1% | 12.9% | 13.1% | 11.9% |
| Outreach visit costs | | 15.0% | 16.4% | 16.5% | 16.9% | 15.0% |

References

1. Berndt, ER and Wood, D: **Technology, prices and the derived demand for energy.** *Rev Econ Stat* 1975, LVII(3): 259-268.

2. Diewert, WE: Separability and a generalization of the Cobb-Douglas cost, production and indirect utility functions, 1973, mimeo. Available at: <http://faculty.arts.ubc.ca/ediewert/cobb.pdf>, accessed 18 December 2012.

3. Kumbhakar SC. **Modeling allocative inefficiency in a translog cost function and cost share equations: An exact relationship**. *J of Econometrics* 1997, 76(1-2): 351-356.

4. Deaton A and Muellbauer J: *Economics and Consumer Behavior*. Cambridge: Cambridge University Press; 1980.
